# Perisynaptic GABA Receptors The Overzealous Protector

**DOI:** 10.1155/2012/708428

**Published:** 2012-02-22

**Authors:** Andrew N. Clarkson

**Affiliations:** Departments of Anatomy and Psychology, University of Otago, P.O. Box 913, Dunedin 9013, New Zealand

## Abstract

An attempt to find pharmacological therapies to treat stroke patients and minimize the extent of cell death has seen the failure of dozens of clinical trials. As a result, stroke/cerebral ischemia is the leading cause of lasting adult disability. Stroke-induced cell death occurs due to an excess release of glutamate. As a consequence to this, a compensatory increased release of GABA occurs that results in the subsequent internalization of synaptic GABA_A_ receptors and spillover onto perisynaptic GABA_A_ receptors, resulting in increased tonic inhibition. Recent studies show that the brain can engage in a limited process of neural repair after stroke. Changes in cortical sensory and motor maps and alterations in axonal structure are dependent on patterned neuronal activity. It has been assumed that changes in neuronal excitability underlie processes of neural repair and remapping of cortical sensory and motor representations. Indeed, recent evidence suggests that local inhibitory and excitatory currents are altered after stroke and modulation of these networks to enhance excitability during the repair phase can facilitate functional recovery after stroke. More specifically, dampening tonic GABA inhibition can afford an early and robust improvement in functional recovery after stroke.

## 1. **γ**-Aminobutyric Acid (GABA)

GABA is the major inhibitory neurotransmitter within the mammalian brain. Twenty to 50% of all synapses within the CNS use GABA as a neurotransmitter, mediating both fast and slow inhibitory synaptic transmission [1]. GABA is an endogenous ligand for the GABA_A_, GABA_B_, and GABA_C_ receptors [2], and these receptor subtypes have been classified according to differences in both structure and pharmacology. GABA_A_Rs are ligand-gated chloride channels [2, 3] formed from 5 subunits arranged around a central ion pore. At least nineteen mammalian genes encoding for the various GABA_A_R subunits exist: *α*_1–6_, *β*_1–3_, *γ*_1–3_, *δ*, *ε*, *φ*, *π*, and *ρ*_1–3_, with slice variants also contributing to variations in receptor functions [4–9]. The most common subunit combinations are believed to be composed of 2*α*, 2*β*, and *γ*, with the *γ*-subunit being able to be substituted for either an *ε*- or a *δ*-subunit [7–9]. 

Depolarization of inhibitory interneurons produces a phasic release of GABA and inhibition of postsynaptic neurons. Extrasynaptic GABA_A_R's respond to ambient levels of GABA present in the extracellular space to regulate baseline pyramidal neuron excitability and show reduced desensitization remaining active for long periods of time [10]. Tonic GABA_A_R's in the hippocampus and cortex contain either *α*5 or *δ*-subunits [6, 10]. Reduced activity of *α*5 or *δ*-subunits enhances pyramidal neuron firing to afferent inputs [10–12], enhances neuronal network excitability [13], and facilitates LTP and cognitive performance [14–17]. GABA transporters modulate the level of tonic GABA_A_R activity [18] with the uptake of GABA into neurons and astrocytes for recycling. Low GABA concentrations activate extrasynaptic GABA_A_R's, leading to persistent or tonic inhibition [19, 20]. Synaptic and extrasynaptic GABA_A_R's exhibit distinct pharmacological and biophysical properties that differentially influence brain physiology and behavior [19].

Synaptic GABA_A_R's are composed of *α*_1–3_, *β*_1–3_, and *γ*_1–3_, subunits, and the site of action for a variety of clinically important drugs, such as benzodiazepines, neurosteroids, and anesthetics. Where as extrasynaptic GABA_A_R's are composed of subunit combinations containing *α*_4–6_, *β*_1–3_, and *γ*_2_- or *δ*-subunits. Of these receptors, the *δ*-containing GABA_A_R's coassembled as *α*_4_ *βδ*—located in the cortex, hippocampus and thalamus—or *α*_6_ *βδ*—located in the cerebellum—that are emerging as unique and fundamental players in GABAergic neurotransmission [19]. In addition to *δ*-containing GABA_A_R's having a functional role in the cortex, the *α*5-containing GABA_A_R's coassembled primarily as *α*_5_ *βγ*_2_ have also been implicated in poststroke repair [21]. Even though the expression of the *α*5-subunit is low in the cortex compared to the *δ*-subunit, greater functional improvements in motor recovery are seen following modulation of the *α*5-subunit [21]. The pharmacology of these extrasynaptic receptors is inconsistent between research groups [22] and has been hampered by the lack of selective agents to probe function in recombinant, native, and whole animal systems [23]. Conflicting data is also present with respect for the ability of these receptors to desensitize [19, 24]. Determining the composition and pharmacology of this receptor will enable the development of much needed therapies for use in stroke.

### 1.1. Disability in Stroke

Stroke is the leading cause of death and long-term disability in adults worldwide. Stroke-induced sensory and motor loss of limb function, in particular, prevents patients from returning to work and accounts for the statistic that almost one-third of stroke survivors become institutionalized after having a stroke [25–28]. Recent studies have shown that the brain has a limited capacity to repair after stroke. In both humans and animals, neural repair after stroke has been shown to involve remapping of cognitive functions and sprouting of new connections in tissue adjacent to the stroke site, the peri-infarct cortex [29, 30]. However, mechanisms associated with poststroke neural repair and recovery have not been well characterized, and it has been assumed that changes in cortical representational maps underlying the recovery involve changes in neuronal excitability. Consistent with this, animal studies suggest that therapies associated with rehabilitation can promote plasticity changes in tissue that survives the stroke [31].

Functional recovery within the peri-infarct cortex involves changes in neuronal excitability. Clinical studies using direct current stimulation of the peri-infarct cortex, with protocols that boost local neuronal excitability, have been shown to improve use of the affected limb in stroke patients [32, 33]. Furthermore, forced use or task-specific repetition of the affected limb have also been shown to activate the peri-infarct cortex and improve functional recovery [34]. Studies suggest that decreases in *γ*-aminobutyric acid GABA activity within the motor cortex could facilitate structural changes [35] and promote recovery of motor function [36]. Alterations in neuronal excitability underlie fundamental changes in information transfer in neuronal circuits [37] such as long-term potentiation and depression (LTP and LTD) as well as the unmasking of quiescent synaptic connections and remodeling of cortical maps [38]. Furthermore, changes in LTP and cortical map formation occur within the peri-infarct cortex adjacent to the stroke [29]. These data suggest a critical role for modulating cortical excitability as a means for promoting functional recovery after stroke.

### 1.2. Brain Excitability in Learning, Memory, and Repair

The processes of neurorehabilitation involve physical, occupational, and cognitive therapies [27, 28]. Further changes in poststroke cortical plasticity play a critical role in mediating repair mechanisms. While these modalities clearly promote functional recovery, no drug treatments exist that promote poststroke brain repair and recovery. Recent evidence suggests that suppression of either cortical tonic GABA inhibition or stimulation of *α*-amino-3-hydroxy-5-methyl-4-isoxazolepropionic acid (AMPA) receptor currents can promote poststroke function gain [21, 39]. This ability to regain function relies heavily on the ability to learn or relearn after stroke and likely follows classical activity-dependent processes associated with motor learning and memory [40, 41]. In addition to these behavioral links, stroke recovery and classical learning and memory pathways share similar molecular and cellular links. For instance, genes that are important for learning and memory are also elevated during periods of poststroke repair and include membrane-associated phosphoproteins GAP43 and MARCKS, the transcription factor c-jun, and the cell adhesion molecule L1 [42].

Modulation of learning and memory pathways have previously been shown to promote functional recovery and poststroke axonal sprouting following administration of pharmacological agents such as amphetamines and phosphodiesterase type-4 inhibitors that boost cAMP/CREB signaling and learning and memory function [43, 44]. These data indicate that manipulating learning and memory pathways can offer a novel means for promoting recovery. As with stroke recovery, the processes of learning and memory can be enhanced by manipulations that increase neuronal excitability, which has also been shown to promote function recovery [21]. Significant data is accumulating indicating an imbalance in inhibitory and excitatory pathways after stroke, and modulation of these pathways by either enhancing glutamate-mediated transmission or dampening the tonic form of GABA can facilitate functional recovery [21, 39, 45–48]. *α*5GABA_A_R negative allosteric modulators are part of a broad class of drugs that boost learning and memory function by influencing key elements in neuronal memory storage, such as LTP [14, 16]. *α*5GABA_A_R negative allosteric modulators, and indeed any mechanism that dampens tonic GABA signaling, could significantly improve poststroke recovery [21]. This suggests that the similarities between neuronal mechanisms of learning and memory and those of functional recovery after stroke extend to common treatment strategies for both.

Most strategies that promote functional recovery after stroke, such as axonal sprouting, neurogenesis, or angiogenesis, focus or rely on inducing structural changes in the brain as a means to promote functional recovery after stroke [49–53]. In order to promote structural change in the brain, however, these treatments take time to develop a functional effect. Blocking tonic GABA inhibition induces a rapid improvement in behavioral recovery in the absence of any change in axonal sprouting within the peri-infarct cortex [21]. This data suggests that treatments that focus on inducing molecular memory systems after stroke may have the advantage of promoting synaptic plasticity in peri-infarct cortex rapidly and without altering the tissue reorganization that normally occurs after stroke. These therapies are highly translatable into the clinic due to their timing of drug administration, 3–7 days after stroke in rodents, and with the early effects seen with functional recovery, will aid in the huge social and economical burdens seen after stroke.

### 1.3. Attenuating GABAA Receptor Function in Neural Repair after Stroke

As with stroke recovery, the processes of learning and memory can be enhanced by manipulations that increase neuronal excitability. However, unlike the stroke recovery field, basic science studies in learning and memory have defined specific cellular pathways that lead to enhanced neuronal excitability and improved function.

Recent work has shown that enhanced neuronal excitability occurs following the dampening of the baseline level of inhibition in neurons. This baseline inhibition is in part set by a tonic, always present, degree of inhibitory signaling from the major inhibitory neurotransmitter, GABA. Unlike the phasic nature of synaptically released GABA, the action of GABA via extrasynaptic receptors is to tonically suppress neuronal excitability and to help regulate neuronal action potential firing. These extrasynaptic GABA receptors consist of *α*5 and *δ*-subunit containing GABA_A_R's. Recent evidence using *α*5GABA_A_R “knock-out”, and point-mutated mice have clearly shown that the *α*5-subunit plays a key role in cognitive processing [15, 17]. In addition, *in vitro* and *in vivo* work has shown that *α*5GABA_A_R negative allosteric modulators can enhance cognition within the Morris water maze, enhance hippocampal LTP and do not have any proconvulsant effects [14, 16]. Using pharmacological and genetic manipulations of extrasynaptic GABA_A_R's, we have shown marked improvements in functional recovery when starting treatments from 3 days after the stroke [21]. These data are consistent for offering a potential role for extrasynaptic GABA_A_R's in processes involving synaptic plasticity and learning and memory and more recently poststroke recovery.

Neuronal inhibition and network function is disturbed in peri-infarct tissue during periods of cortical plasticity, re-mapping, and recovery. The increase in tonic inhibition in cortical pyramidal neurons reported by Clarkson and colleagues [21] occurs at precisely the same time as cortical map plasticity and recovery [54]. Behavioral recovery in stroke is closely correlated with functional plasticity in peri-infarct and connected cortical regions. In human stroke patients, an expansion in motor representation maps is seen in tissue adjacent to or connected to stroke [29, 55]. In animal models, when stroke damages primary motor or somatosensory areas, motor and sensory representations remap in peri-infarct cortex [54, 56]. These processes of recovery identify plasticity in the cortical circuits in peri-infarct cortex as key elements in functional recovery.

## 2. GABA and Cerebral Ischemia

A large body of work has been devoted to developing and exploring neuroprotectants that act to block glutamate-mediated neurotransmission in animal models of cerebral ischemia [57, 58]. Increased inhibitory neurotransmission associated with GABA has been shown to normalize the balance of glutamate-mediated excitation. Therefore, pharmacological enhancement of GABA_A_R neurotransmission provides an alternative means for neuroprotection. Indeed, over recent years, changes in GABA function following cerebral ischemia and possible protective benefits of GABAergic drugs have been extensively assessed [59–65]. Even though it has been proposed that enhancing GABA transmission may elicit protection against cerebral ischemia [60–62, 65], the exact mechanisms that are associated with these neuroprotectants have, as yet, not been fully elucidated and increasing GABA function may be protective during cerebral ischemia for different reasons [59–65]. However, even though GABA agonists have shown great promise in animal model, these compounds have failed to translate into the clinic [66, 67]. The failure of these compounds highlights the need to firstly establish better preclinical rodent models of stroke that better mimic what occurs in humans. Secondly, the use of subunit specific GABA compounds is more likely to show an effect, due to them having less side effects, such as drug-induced hypothermia and sedation. However, even with recent developments in this area, studies are lacking. The need to assess subunit-specific GABA compounds to help understand what is happening after stroke in terms of GABA function is highlighted with clinical reports showing that zolpidem, an *α*1 subunit GABA_A_R modulator, can result in transiently improves in aphasia in chronic stroke survivors [68].

During situations of cerebral ischemia, it has been shown that the extracellular concentrations of GABA increase (approx. 50 fold compared to basal levels) to the micromolar range [59, 69] and remain elevated for at least 30 minutes during periods of reperfusion. Prolonged exposure of the GABA_A_Rs to high concentrations of GABA agonists *in vitro* has routinely been shown to become desensitized and/or downregulated [70–72]. Similarly, the GABA_A_R is also downregulated in the gerbil hippocampus following transient cerebral ischemia [63]. In this model, receptor downregulation was shown to be via internalization, as there was a rapid decrease in binding of the hydrophilic ligand [3H]-SR-95531, but not the hydrophobic ligand [3H]-flunitrazepam [63]. This increase in extracellular GABA is likely to result in the spill over onto peri-synaptic GABA_A_R's resulting in an increase in tonic inhibition. Indeed, recent evidence showing an increase in tonic inhibition after stroke supports this notion [21]. This increase in tonic inhibition is most likely a safety mechanism imposed by the brain as a means to minimize neuronal damage. However, as this increase in tonic inhibition persists for at least 2 weeks after the stroke, this safety mechanism which is likely to have either wrong or no feedback mechanism has been formed to compensate for such a change in tonic GABA.

## 3. Poststroke Tonic Inhibition

Changes in neuronal excitability, loss of GABAergic inhibition, enhanced glutamatergic transmission, and synaptic plasticity all contribute to neuronal reorganization after stroke. Studies that promote an increase in local brain excitability result in improved function [21, 34, 39, 45] and suggest that decreasing GABA activity within the brain could facilitate structural changes that promote functional recovery [21, 34, 45]. In particular, this enhancement of neuronal excitability involves dampening baseline levels of inhibition.

Tonic or continuous signaling from GABA sets baseline inhibition. GABA acts via extrasynaptic GABA_A_R's to tonically suppress neuronal excitability and regulate neuronal action potential firing. Therefore, in order to facilitate functional recovery, an increase in brain excitability is required to overcome this hypofunctionalism [34]. Recently Clarkson and colleagues have demonstrated marked improvements in poststroke functional recovery using pharmacological manipulations of extrasynaptic GABA_A_R's, implicating *α*5 or *δ*-containing GABA_A_R's as novel targets for developing agents to help stroke sufferers.

GABA has been shown to mediate both fast and slow inhibitory synaptic transmission [1]. During development, however, the GABA_A_Rs have been shown to mediate excitation as well as play an important role in neural migration and synaptogenesis [73, 74]. During situations of cerebral ischemia, extracellular concentrations of GABA are significantly elevated [59, 69], resulting in GABA_A_ receptor desensitization and/or downregulation [63, 71]. This is supported by immunohistochemical and autoradiographic data showing decreased expression of *α*1, *α*2, *α*3, *α*5, and *γ*2 subunits following photothrombotic stroke and freeze-lesion-induced cortical injury [75–77].

Recent work has shown that epileptogenesis results in the suppression of functionally active *α*5GABA_A_Rs and results in an increase/substitution of other GABA_A_R's with a subsequent increase in rather than suppression of tonic inhibitory currents [78]. A similar compensatory increase in *α*4*δ*-mediated tonic currents has been seen in the *α*5 knockout mice within region CA1 of the hippocampus [11]. Extracellular GABA concentrations and thus tonic inhibition have been shown to increase as the excitatory drive increases resulting in the modulation of neuronal excitability and prevention of neuronal saturation [79]. Consistent with these findings, Clarkson and colleagues reported an increase in GABA tonic inhibitory currents from 3–14 days poststroke in layer II cortical pyramidal neurons [21]. This poststroke increase in tonic inhibition may act as a compensatory mechanism to prevent further neuronal injury. However, this prolonged increase in tonic inhibition during the repair phase is acting as a hindrance by preventing cortical expansion and improvements in functional recovery. This is supported by findings by Clarkson and colleagues who show that both pharmacological and genetic modulation of tonic inhibition, dampening either *α*5 or *δ*-mediated increase in tonic GABA currents, results in early and marked improvements in functional recovery [21].

Understanding the profile for which cortical plasticity occurs, altered after a stroke, is critical for fully determining when to start treatments and with what therapeutic compound to use. Based on our findings, we have clearly shown that dampening of tonic GABA currently from 3 days results in robust functional improvements of motor recovery [21]. These improvements, however, may not be the same if treatments are started weeks after stroke onset as previously shown in humans using zolpidem, which was shown to transiently improve aphasia in chronic stroke survivors [68]. The *α*1 and *β*2 GABA_A_R subunits are densely localized within the cortex and coassembly with the *γ*2-subunit accounts for about 40% of all GABA_A_Rs within the cortex [80]. Assembly of GABA_A_Rs containing *α*1*β*2*γ*2 has been shown to be enriched at synaptic sites throughout the cortex [81] and involved in changes in synaptic plasticity. However, studies have also shown that the *δ* subunit can coassemble with *α*1 subunits to form functional recombinant receptors[82, 83]. Furthermore, immunoprecipitation studies have shown that *δ* subunits can associate with *α*1 subunits [84], and GABA_A_R*α*1 subunits have also been found extrasynaptically [85, 86] consistent with the typical localization of *δ*-containing GABA_A_Rs^8181^. These data could suggest an alternative method for why zolpidem was having an effect in chronic stroke patients to alleviate the burden of aphasia. However, further studies are needed, as one previous study would suggest that the *γ*2-subunit is required in order for zolpidem to have an effect [87].

## 4. Dampening Cortical Inhibition Alters Cortical Responsiveness

Disinhibition of cortical connections within the peri-infarct or regions associated with the peri-infarct cortex have been argued as either occurring as a direct consequence of the stroke or as a potential compensatory mechanism related to the recovery [88]. This argument has come about based on a number of observations such as local blockage of GABAergic inhibition unmasking preexisting horizontal connections within the rat motor cortex [38]; LTP of adult rat motor cortex horizontal connections is dependent on GABA disinhibition during theta burst stimulation, unlike other regions such as the hippocampus or somatosensory cortex [35]; and finally modulation of GABA has been shown to be involved in learning in healthy humans as shown using imaging studies showing a correlation between a decrease in GABA concentration in motor cortex and motor skill learning [89]. Consistent with the notion that cortical disinhibition is occurring as a compensatory mechanism, Clarkson and colleagues have shown a robust and persistent increase in tonic inhibition in the peri-infarct cortex after stroke and blockade of this tonic inhibition at the time of stroke with the extrasynaptic GABA_A_R negative allosteric modulator, L655-708, exacerbated the lesion [21]. Further to this, Clarkson and colleagues showed for the first time that delayed treatment L655-708, which has previously been shown to induce LTP [14], provides an early and robust reversal in behavioral deficits [21]. Given the early behavioral effects seen and the lack of effect on sprouting of new connections, cortical disinhibition following L655-708-treatment seems a logical argument. To support the notion that dampening GABA activity is having a beneficial effect, no improvement in motor function was observed after stroke following administration of the GABA agonist, muscimol [21]. This is backed by clinical studies illustrating the reemergence of stroke symptoms following administration of the GABA agonist midazolam in chronic stroke patients that have shown significant improvements in function [90]. The peri-infarct cortex exhibits neuronal metabolic dysfunction over a one-month period [91], which would indicate a therapeutic time window for blockade of tonic GABA signaling of at least one month after stroke. Consistent with this is the fact, when L655-708 treatment is discontinued after a two-week period of administration after stroke, a slight rebound effect/reversal in functional recovery is observed compared to animals that received treatment for the six-week period [21].

## 5. Conclusions

Therapies that promote functional recovery after stroke are limited to physical rehabilitation measures. While specific measures, such as constraint-induced therapies, promote recovery of motor function, no pharmacological therapies are available that aid in recovery. Functional recovery after stroke follows psychological learning rules [41] that indicate learning and memory principles may underlie behavioral recovery. At the cellular level, learning and memory are mediated by specific excitatory neuronal responses, such as LTP, and are potentiated by drugs that facilitate aspects of excitatory neuronal signaling [13], such as tonic GABA_A_R antagonists [10]. Recent data shows that stroke alters the balance of excitatory and inhibitory inputs to neurons in the peri-infarct cortex, by increasing inhibitory tone. This altered excitatory balance occurs through a decrease in the normal cellular uptake of GABA. Dampening GABA-mediated tonic inhibition restores the excitatory/inhibitory balance in peri-infarct motor cortex *ex vivo* and promotes recovery of motor function *in vivo*. These effects occur through blockade of *α*5 or *δ*-containing GABA_A_R's. This data indicates a novel role for tonic GABA_A_R function in promoting poststroke recovery most likely via cortical disinhibition [38, 92, 93] and suggests a new avenue for pharmacological treatment of neurorehabilitation in stroke. This early effect on stroke recovery opens the possibility for treatments that block tonic GABA signaling and may be used in conjunction with later-acting stroke repair therapies in a combinatorial manner. More generally, tonic GABA signaling has a biphasic role in stroke. Early tonic GABA signaling limits stroke size, later tonic GABA signaling limits stroke recovery. These data identify a promising molecular system for future stroke recovery therapies and implicate molecular memory systems as likely key players in recovery from stroke.
